# Disseminated Juvenile Xanthogranuloma in an Infant Requiring Systemic Therapy: A Case Report

**DOI:** 10.7759/cureus.108031

**Published:** 2026-04-30

**Authors:** Emelie Simone Okouango, Bouchra Baghad, Hanane Rachadi, Soumiya Chiheb

**Affiliations:** 1 Department of Dermatology, Ibn Rochd University Hospital Center, Faculty of Medicine and Pharmacy of Casablanca, Hassan II University, Casablanca, MAR

**Keywords:** corticosteroids, disseminated juvenile xanthogranuloma, immunohistochemistry, infant, non-langerhans cell histiocytosis

## Abstract

Juvenile xanthogranuloma (XGJ) is a generally benign non-Langerhans cell histiocytosis (non-LCH) that primarily affects infants and children. It typically presents as a single skin lesion. Disseminated and profuse forms remain rare and can pose diagnostic or therapeutic challenges. The classification of histiocytoses has recently evolved, incorporating disseminated XGJ into group “C” of non-LCH cutaneous histiocytoses. While the prognosis for isolated cutaneous forms is excellent, vigilance regarding potential associations (type 1 neurofibromatosis and juvenile myelomonocytic leukemia) and systemic complications remains essential. We report the case of a four-month-old infant presenting with a purely cutaneous disseminated form of XGJ, confirmed by histopathology and immunohistochemistry, which required systemic corticosteroid therapy due to the rapid spread of the lesions. This study involves a four-month-old infant with no significant family history who has been presenting with gradually progressive papular skin lesions for one month. Clinical examination revealed multiple umbilicated, yellowish-brown papulonodules scattered across the face, trunk, limbs, skin folds, genital mucosa, and scalp; a café-au-lait spot was detected on one limb. Dermoscopy revealed a characteristic “sunset” pattern. Histopathological and immunohistochemical analysis (CD68+, CD163+, CD1a-, CD34-, S100-) confirmed the diagnosis. The staging evaluation revealed no abnormalities. After three months of simple monitoring, the extensive spread of the lesions prompted the initiation of oral corticosteroid therapy (1 mg/kg/day), resulting in partial regression at six weeks. Disseminated XGJ in infants remains a rare condition. Although the course is most often self-limiting, justifying an initial conservative approach, certain extensive forms may require systemic treatment. The dermatologist plays a central role in diagnosis, based on a combination of clinical, dermoscopic, histopathological, and immunohistochemical findings, and in the multidisciplinary decision-making regarding treatment.

## Introduction

Juvenile xanthogranuloma (XGJ) is a proliferative histiocytic disorder of dermohypodermic dendritic origin [1], belonging to the group of non-Langerhans cell histiocytoses (non-LCH) [2]. It occurs primarily in early childhood before the age of 2, with a marked male predominance, and its incidence is estimated at one case per one million children [1]. Typically benign and self-limiting, it most often presents as a single or multiple yellowish-brown cutaneous nodules measuring 0.5-1 cm in diameter, predominantly located in the cephalic region (head or neck), and disappears spontaneously within a few months to a few years [3]. The rarer disseminated, profuse forms are characterized by multiple skin lesions (more than five) and may be associated with mucosal and systemic involvement (eye, lung, liver, and central nervous system) [3]. In addition, the concurrent occurrence of neurofibromatosis type 1 (NF1), juvenile myelomonocytic leukemia, and XGJ has also been described [4]. The recent classification of histiocytoses has clarified the place of disseminated XGJ in Group C among non-LCH proliferations, highlighting the diagnostic value of immunophenotyping (CD68+, CD163+, Factor XIIIa+, CD1a-) [5]. We report the case of a four-month-old infant presenting with a purely cutaneous disseminated form of XGJ, with an initially extensive course, in order to discuss its diagnostic features.

## Case presentation

**Figure 1:**
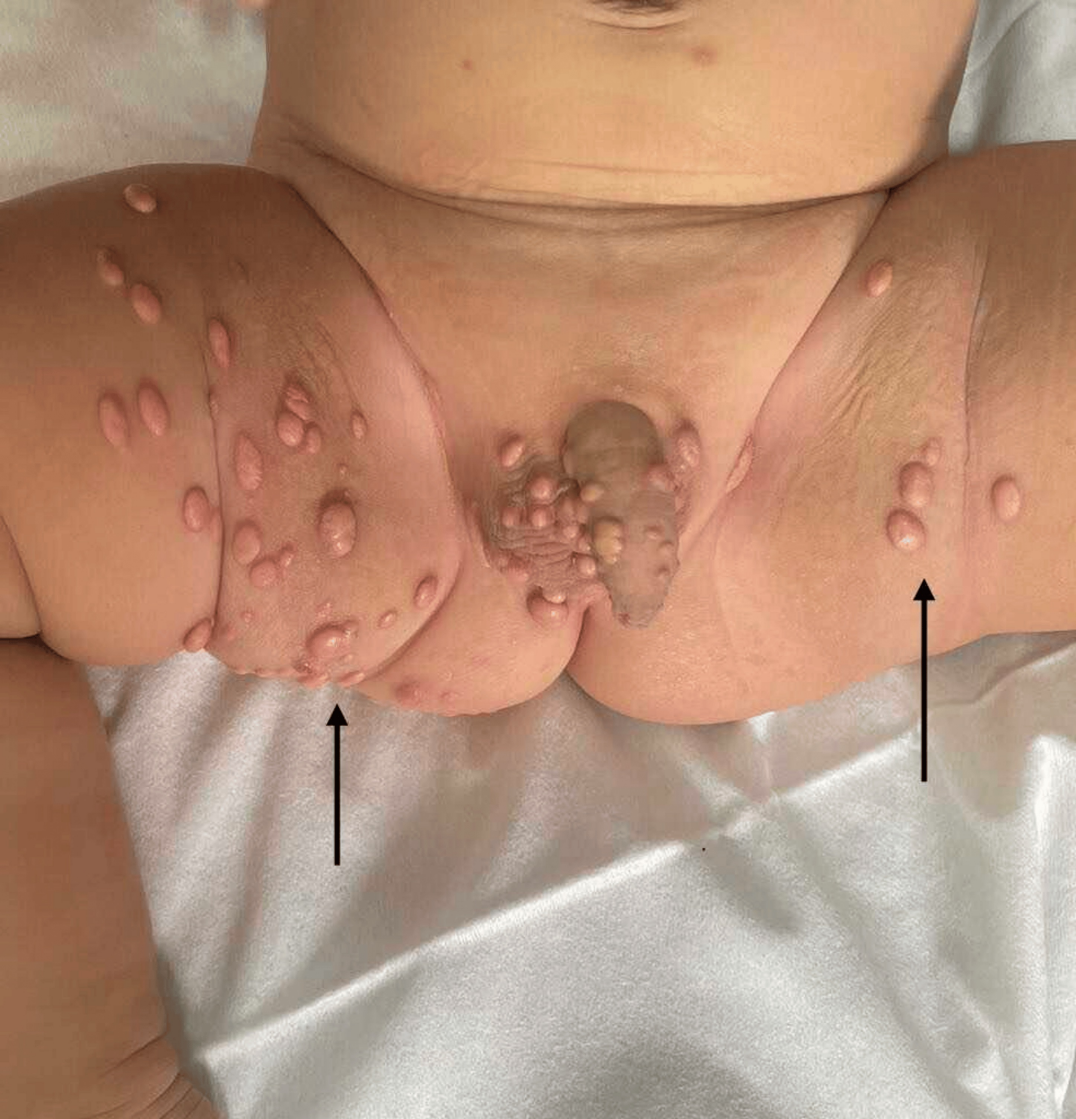
Dermatological examination showing brownish papulonodular lesions, flat-topped or dome-shaped, with an umbilicated center on the genital region

This study is about a four-month-old male infant born at term via uncomplicated vaginal delivery, the third of three children born to nonconsanguineous parents, with good psychomotor development and no significant family history, who has presented for the past month with skin lesions described as papular, initially located in the genital area, and subsequently spreading to the trunk, armpits, and neck. No triggering factors were identified. All of this occurred in the context of normothermia and stable general condition. Dermatological examination revealed multiple brownish papulonodular lesions, flat-topped or dome-shaped, with an umbilicated center in some areas. These lesions, which were firm, indurated, and painless, measured between 1 and 3 cm in diameter. They were scattered across the face (forehead, right upper eyelid, left ear), the trunk (right lateral thoracic region, back), the limbs (inner thighs, anterior and posterior surfaces of the left leg), skin folds (neck, right axilla, inguinal regions, retroauricular regions), the anteromedial aspect of the sole of the right foot, the genital region (buttocks, perineum, scrotum, penis, urethral meatus), and the scalp (Figure 1). The oral and ocular mucosae, as well as the palmar region, were spared.

**Figure 2:**
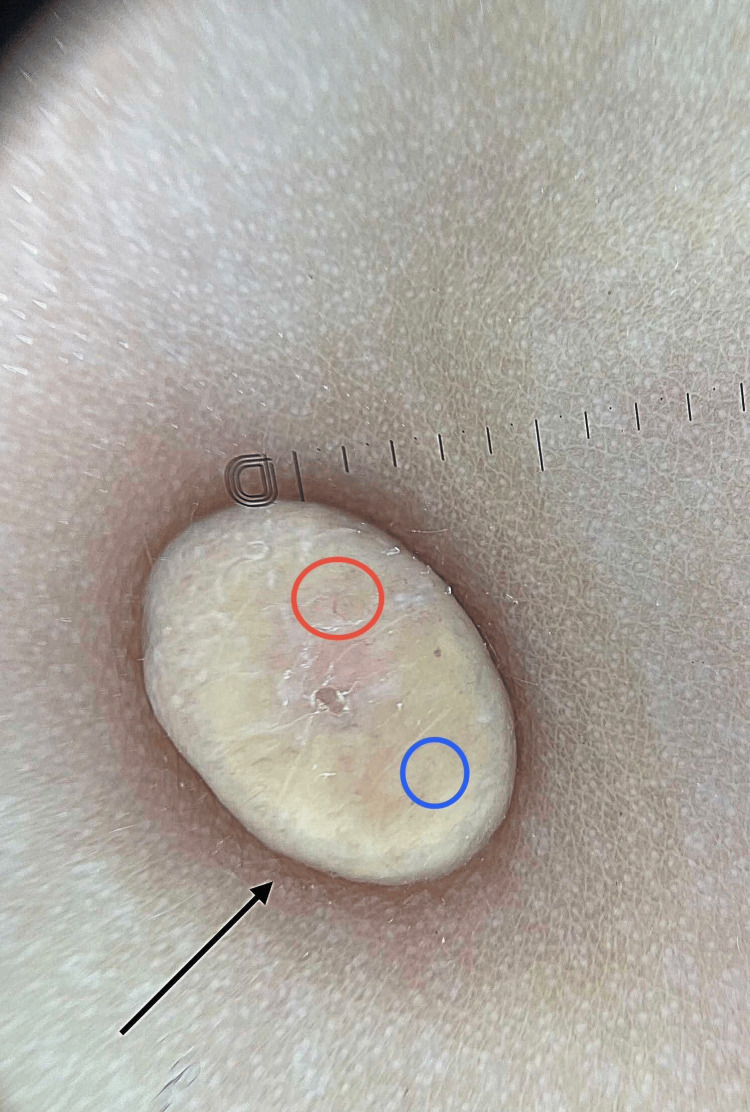
Dermoscopic appearance showing the “sunset” pattern (blue circle: central yellowish clusters; red circle: peripheral vascularization)

Dermoscopy (Heine Delta 20T® Dermatoscope, HEINE Optotechnik GmbH & Co. KG, Gilching, Germany) performed on several lesions revealed a lichenoid structure with central yellow-orange clusters and a peripheral “sunburst” vascular network, corresponding to the classic “sunset” pattern (Figure 2).

**Figure 3:**
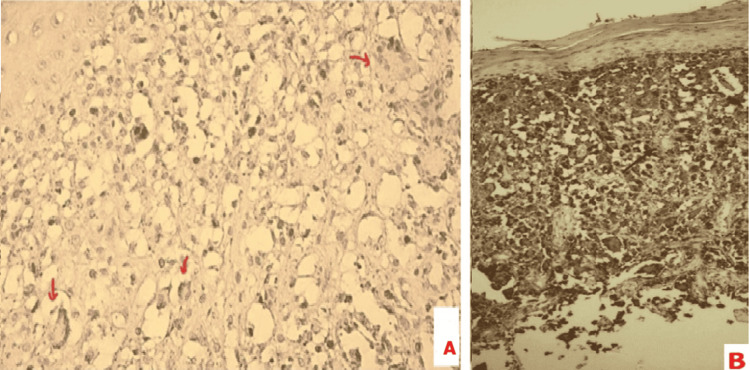
(A) Histopathological examination showing dermal infiltrate with Touton giant cell (arrow) (H&E, x40). (B) Immunohistochemical analysis showing positive immunohistochemical staining for CD68 (IHC, x10) IHC: immunohistochemistry; H&E: hematoxylin and eosin

A skin biopsy of the lesion was performed. Histopathological examination revealed a dense dermal infiltrate composed of foam cells and multinucleated giant cells of the Touton type, without cytonuclear atypia (Figure 3A). Immunohistochemical analysis (Figure 3B) revealed strong expression of CD68 and CD163 by the histiocytic cells, which were, however, negative for CD1a, CD34, and S100 protein. These findings confirmed the diagnosis of XGJ.

The main differential diagnoses were Langerhans cell histiocytosis, mastocytosis, and molluscum contagiosum. The systemic evaluation was normal; no systemic abnormalities were found. A comprehensive evaluation, including a complete blood count, liver function tests, a full ophthalmological examination, a chest X-ray, and an abdominal ultrasound, showed no abnormalities.

**Figure 4:**
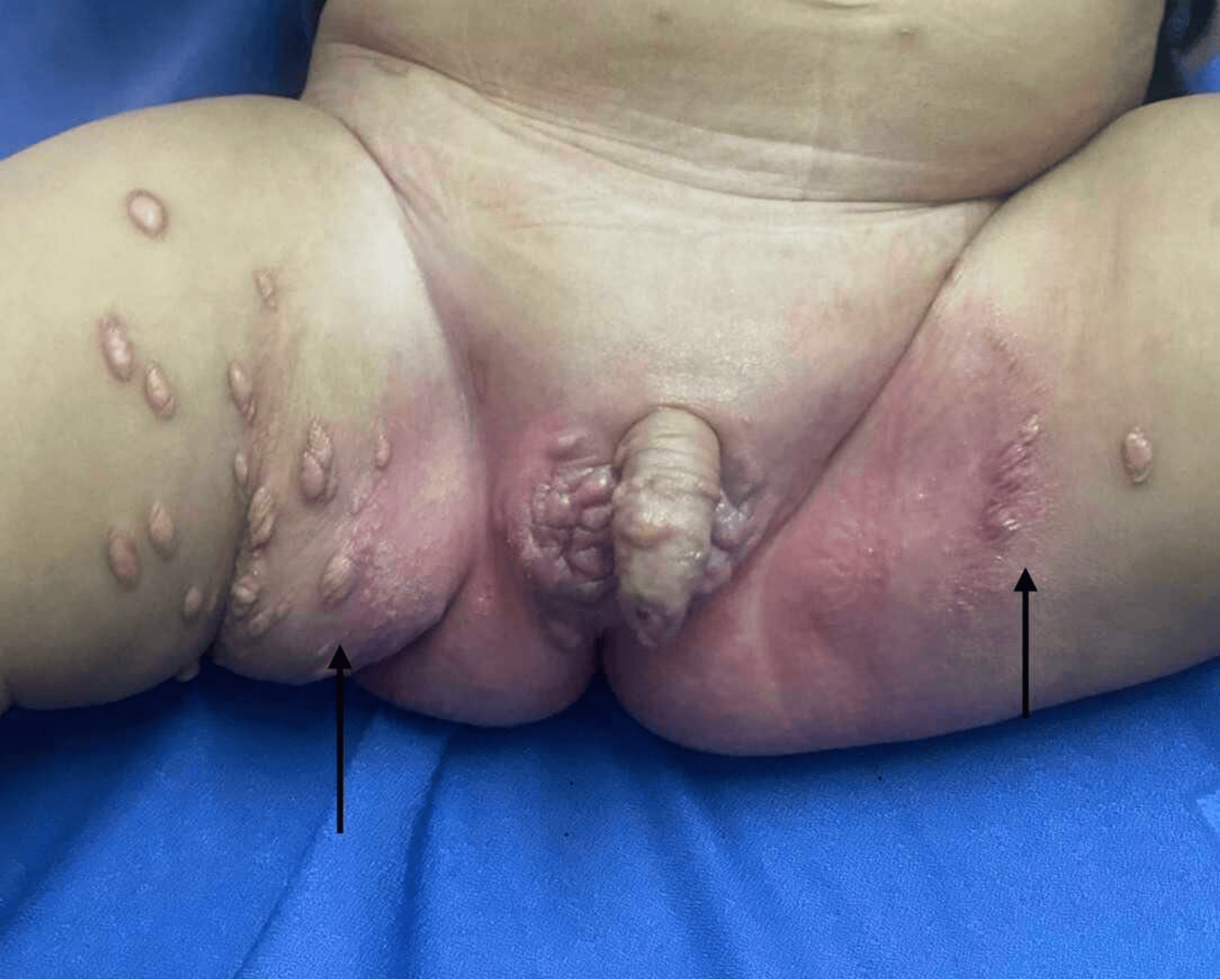
Evolution showing partial regression and atrophy of lesions after six weeks of corticosteroid therapy

Given the purely cutaneous nature of the disease, the multiple lesions, and the favorable prognosis, simple monitoring was initially instituted for three months. Given the extensive spread of the lesions and the appearance of a lesion at the urethral meatus, treatment with oral corticosteroids (prednisone, 1 mg/kg/day) combined with adjuvant therapy (calcium supplementation) and close clinical monitoring was initiated. The course of the disease at six weeks of treatment was favorable, marked by the absence of new lesions and the regression and atrophy of preexisting lesions (Figure 4).

## Discussion

XGJ results from a reactive macrophage proliferation, likely triggered by an unidentified inflammatory stimulus [1]. It most commonly appears in infants as a single papule or nodule, regressing spontaneously within one to five years [3]. The clinical presentation of multiple lesions with a diffuse distribution is typical, although rare [5].

Dermoscopy has been a valuable diagnostic tool. The “sunset” pattern, due to the presence of lipid-laden foam cells and reactive neovascularization, is highly suggestive of the diagnosis in the early stages [6]. This is a typical dermoscopic feature; regardless of ethnic background, it is similar in both fair-skinned and dark-skinned children. However, histology and immunohistochemistry remain essential for confirming the diagnosis and ruling out Langerhans cell histiocytosis, which has a different prognosis and management [6]. The immunohistochemical profile (CD68+/CD163+/CD1a-/S100-) is characteristic of the xanthogranuloma group [4,6].

Management of cutaneous-only forms is typically conservative, as the majority of lesions resolve spontaneously within three to five years [2,7]. In cases of multiple and extensive lesions, various treatments have been reported: systemic corticosteroid therapy, mitogen-activated protein kinase kinase inhibitors, vinblastine, or interferon α in cases of life-threatening systemic involvement [8,9]. However, as in our case, a minority of disseminated forms may exhibit proliferative progression or cause functional or cosmetic distress, warranting treatment. Systemic corticosteroid therapy is a valid first-line treatment option in these situations, often allowing for control of lesion progression [8,10].

Our case report illustrates a disseminated, purely cutaneous form of XGJ in an infant, whose extensive progression and involvement of the urethral meatus warranted systemic treatment. Our patient responded favorably to prednisone, with partial regression and no adverse effects, confirming the value of this treatment in extensive forms.

This case highlights the importance of a thorough initial assessment of disease extent and long-term follow-up, particularly during the first two years, to detect any systemic involvement or the emergence of NF1 or LMMJ [4,7]. Current recommendations favor a rigorous clinical examination and a systematic ophthalmological examination, without extensive imaging in the absence of warning signs [4,10].

## Conclusions

Disseminated XGJ in infants is a rare condition that requires an accurate diagnosis and a thorough evaluation of the extent of the disease, particularly with regard to the eyes. Diagnosis is based on clinical presentation, dermoscopy, and histopathological and immunohistochemical confirmation. While the condition most often resolves spontaneously, our observation shows that certain extensive forms may require systemic treatment, such as oral corticosteroid therapy, with notable efficacy. A multidisciplinary approach involving the dermatologist, pediatrician, and ophthalmologist is essential for optimal management of these patients. This case illustrates the typical clinical presentation, the value of dermoscopy, histopathological and immunohistochemical confirmation, and a good response to treatment.
